# Autologous Matrix Induced Chondrogenesis (AMIC) Compared to Microfractures for Chondral Defects of the Talar Shoulder: A Five-Year Follow-Up Prospective Cohort Study

**DOI:** 10.3390/life11030244

**Published:** 2021-03-16

**Authors:** Filippo Migliorini, Jörg Eschweiler, Nicola Maffulli, Hanno Schenker, Arne Driessen, Björn Rath, Markus Tingart

**Affiliations:** 1 Department of Orthopedics and Trauma Surgery, University Clinic Aachen, RWTH Aachen University Clinic, 52064 Aachen, Germany; migliorini.md@gmail.com (F.M.); joeschweiler@ukaachen.de (J.E.); hschenker@ukaachen.de (H.S.); adriessen@ukaachen.de (A.D.); Bjoern.Rath@klinikum-wegr.at (B.R.); mtingart@ukaachen.de (M.T.); 2 School of Pharmacy and Bioengineering, Keele University School of Medicine, Staffordshire ST4 7QB, UK; 3 Barts and the London School of Medicine and Dentistry, London E1 2AD, UK; 4 Centre for Sports and Exercise Medicine, Queen Mary University of London, Mile End Hospital, London E1 4DG, UK; 5 Department of Orthopedics, Klinikum Wels-Grieskirchen, A-4600 Wels, Austria; 6 Department of Medicine, Surgery and Dentistry, University of Salerno, 84081 Baronissi, Italy

**Keywords:** talus, ankle, chondral defect, autologous matrix induced chondrogenesis, AMIC, microfractures, management, surgery

## Abstract

Introduction: Many procedures are available to manage cartilage defects of the talus, including microfracturing (MFx) and Autologous Matrix Induced Chondrogenesis (AMIC). Whether AMIC or MFx are equivalent for borderline sized defects of the talar shoulder is unclear. Thus, the present study compared the efficacy of primary isolated AMIC versus MFx for borderline sized focal unipolar chondral defects of the talar shoulder at midterm follow-up. Methods: Patients undergoing primary isolated AMIC or MFx for focal unipolar borderline sized chondral defects of the talar shoulder were recruited prospectively. For those patients who underwent AMIC, a type I/III collagen resorbable membrane was used. The outcomes of interest were: Visual Analogic Scale (VAS), Tegner Activity Scale, American Orthopedic Foot and Ankle Score (AOFAS). The Magnetic Resonance Observation of Cartilage Repair Tissue (MOCART) was assessed by a blinded radiologist, who had not been involved in the clinical management of the patients. Data concerning complication rate and additional procedures were also collected. Results: The mean follow-up was 43.5 months. The mean age of the 70 patients at operation was 32.0 years, with a mean defect size of 2.7 cm^2^. The mean length of hospitalization was shorter in the MFx cohort (*p* = 0.01). No difference was found between the two cohorts in terms of length of prior surgery symptoms and follow-up, mean age and BMI, sex and side, and defect size. At a mean follow-up of 43.5 months, the AOFAS (*p* = 0.03), VAS (*p* = 0.003), and Tegner (*p* = 0.01) scores were greater in the AMIC group. No difference was found in the MOCART score (*p* = 0.08). The AMIC group evidenced lower rates of reoperation (*p* = 0.008) and failure (*p* = 0.003). Conclusion: At midterm follow-up, AMIC provides better results compared to MFx.

## 1. Introduction

Focal chondral defects of the talar shoulder are common [1,2]. Given the limited self-healing capability of cartilage, chondral defects are debilitating, often requiring surgical management [3,4,5]. Isolated microfractures (MFx) are recommended for defects smaller than 2.5 cm^2^ [6,7,8,9,10]. For bigger defects, several different surgical techniques have been described [11,12,13,14]. Osteochondral allo- or autograft transplantation (OAT) has been extensively performed as management of such chondral defects [15,16,17]. While autografts require a harvest site, allografts are expensive and have greater risk of failure [18,19]. Autologous chondrocyte implantation (ACI) has been widely used to address talar chondral defects [20,21]. ACI is a two sessions surgery which requires the harvest of cells and external chondrocytes expansion [22,23]. Autologous Matrix-Induced Chondrogenesis (AMIC) has been recently introduced [2,24]. AMIC does not require a harvest site, cell expansion, and is performed in a single surgical session [25,26]. AMIC is combined with MFx covering the lesions with a resorbable membrane to stabilize the resulting blood clot [27,28]. Thus, AMIC exploits the regenerative potential of bone marrow-derived mesenchymal stem cells arising from the subchondral bone [25,29].

Whether AMIC performed better than MFx for borderline sized defects (2.2 to 2.8 cm^2^) of the talar shoulder is unclear. The present study compared the efficacy of primary isolated AMIC versus MFx for borderline sized focal unipolar chondral defects of the talar shoulder at midterm follow-up. We hypothesized that AMIC provides better outcomes compared to MFx.

## 2. Material and Methods

### 2.1. Patients Recruitment

**Figure 1:**
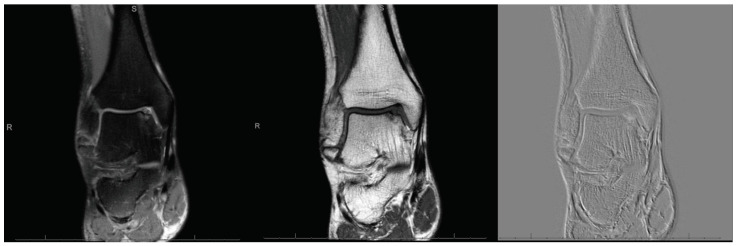
MRI evidencing a focal defect of the medial talar shoulder.

The present study was performed according to Strengthening the Reporting of Observational Studies in Epidemiology: the STROBE Statement [30]. In our setting, for patients with defect sized 2 to 3 cm^2^, both AMIC or isolated MFx were routinely performed. From 2012, patients undergoing primary isolated AMIC or MFx for focal unipolar borderline sized chondral defects of the talar shoulder (Figure 1), were recruited and followed-up prospectively. The inclusion criteria were: (1) symptomatic chondral defect of the talar shoulder, (2) single focal defect sized 2 to 3 cm^2^, (3) MRI evidence, (4) patients able to understand the nature of the treatment and the study. The exclusion criteria were: (1) kissing lesions, (2) bilateral lesions, (3) multifocal lesions, (4) previous ankle surgeries, (5) any bone disease, (6) any skeletal malformation, (7) any other relevant pathology that could have influenced the study. Suitable patients were informed about the pros and cons of both techniques, and were left free to decide their own procedure. The present study was approved and registered by the ethic committee of the RWTH University of Aachen (project ID EK 438-20), and conducted according to the principles expressed in the Declaration of Helsinki. All patients were able to understand the nature of their treatment and provided written consent to use their clinical and imaging data for research purposes.

### 2.2. Surgical Technique

All the surgeries were performed by three surgeons (BR, MT, AD) in a highly standardized fashion. All the surgeons were well behind their learning curve and had no preference on the surgical procedure. Briefly, the ankle was plantar flexed and a 2 mm K-wire was drilled in the distal tibia and another one in the talus. A Hintermann spreader (Integra LifeSciences, Plainsboro, NJ, USA) was used for joint distraction. Lesions were arthroscopically approached through standard anterolateral and anteromedial portals, according to the defect location shown on MRI. After identification of the defect, debridement and curettage of the non-viable border of the chondral tissue surrounding the lesion was performed until viable shoulder cartilage was reached. At this stage, the two surgical techniques take two different turns. In the MFx group, microfractures of 4 mm depth were arthroscopically performed into the defect. In the AMIC group, an arthroscopically-assisted mini-arthrotomy approach was used. A malleolar osteotomy was performed if the defect was not accessible by simple mini-arthrotomy. Microfractures of 4 mm depth were performed into the defect using a 1.2- or 1.4-mm Kirschner wire under constant irrigation with normosaline. If subchondral bone was not viable, this was debrided and substituted with autologous cancellous bone graft harvested from the osteotomy site or from the ipsilateral iliac crest. An aluminum template was trimmed according to the defect. A resorbable porcine type I/III collagen was used in all patients (Chondro-Gide^®^, Geistlich Pharma AG, Wolhusen, Switzerland). The membrane was trimmed according to the aluminum template to be slightly undersized in relation to the defect to avoid displacement, and hydrated in a saline solution. The membrane was placed into the lesion and secured with fibrin glue. The stability of the membrane was checked by flexing and extending the ankle. When an osteotomy was performed, it was fixed with two malleolar screws inserted through the predrilled holes and the wound sutured in a standard fashion. The rehabilitation protocol was performed according to that previously published [25].

### 2.3. Outcomes of Interest

Prior to surgery, the following data were recorded: age, gender, side, area of defect, additional autologous spongiosa transplantation, BMI (Kg/m^2^), score, symptoms duration, prior surgery, and length of the hospital stay. At the last follow-up, patients underwent an MRI and subsequently were invited to answer the following questionnaires: Visual Analogic Scale (VAS), Tegner Activity Scale, American Orthopedic Foot and Ankle Score (AOFAS). The Magnetic Resonance Observation of Cartilage Repair Tissue (MOCART) was assessed by a blinded radiologist, who had not been involved in the clinical management of the patients. Data concerning complications (failure, revision, arthroplasty, delamination, hypertrophy) and additional procedures were also collected. Failure was defined as persistent pain that affected negatively the quality of life and limited participation in recreational activities. For those patients who underwent a malleolar osteotomy, the occurrence of screw removal was not considered as revision surgery.

The primary outcome of interest was to compare the outcomes at the last follow-up between the AMIC and MFx group. The secondary outcome of interests was to compare within AMIC the location of the lesion (medial vs. lateral), the effect of the bone grafting (bone graft vs. no bone graft), and the approach (distraction vs. osteotomy).

### 2.4. Statistical Analysis

All statistical analyses were performed using the software IBM SPSS version 25. Continuous data were analyzed using the mean difference (MD), while for dichotomic data, the odd ratio (OR) effect measures. The confidence interval was set at 95% in all the comparisons. The T-test and x^2^ tests were performed, respectively, with values of *p* < 0.05 considered statistically significant. The confidence interval (CI) was set at 95% in all comparisons.

## 3. Results

### 3.1. Recruitment Procedure

**Figure 2:**
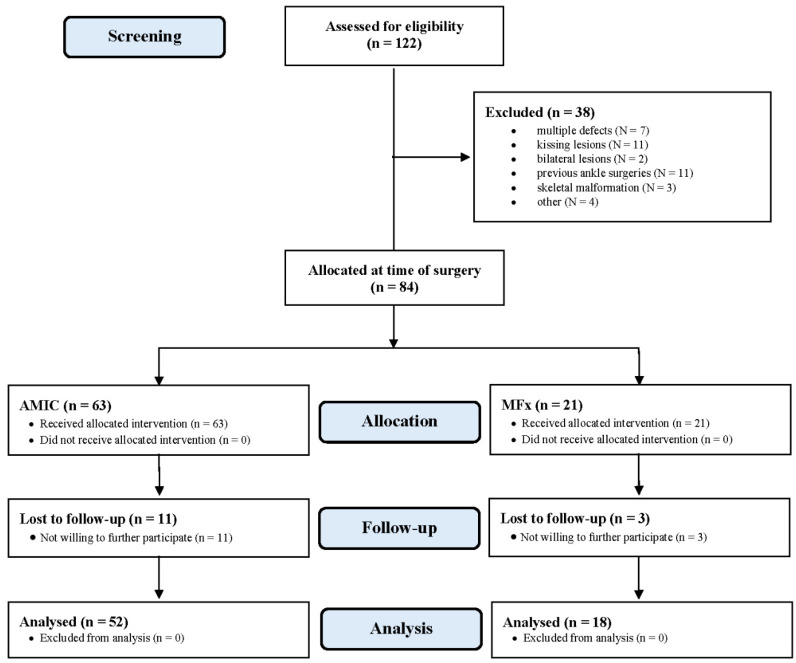
Diagram of the recruitment process (AMIC: Autologous Matrix Induced Chondrogenesis; MFx: Microfracures). Compared to Microfractures.

A total of 122 patients were initially evaluated. Of them, 38 were not eligible: multiple defects (N = 7), kissing lesions (N = 11), bilateral lesions (N = 2), previous ankle surgeries (N = 11), skeletal malformation (N = 3), other (N = 4). This left 84 patients: 63 AMIC and 21 MFx. A further 14 patients were lost to follow-up: 11 patients in the AMIC group, and 3 in the MFx group did not wish to further participate in the study for geographical reasons, but they declared themselves satisfied on telephone interviews. Eventually, 70 patients took part in the present study: 52 in the AMIC group and 18 in the MFx (Figure 2).

### 3.2. Patients Demographics

The mean follow-up was 43.5 months. The mean age of the 70 patients on admission was 32.0 years, with a mean defect size of 2.7 cm^2^, 44% (31 of 70 patients) were women, and in 53% (37 of 70) of patients the right side was involved. The mean symptoms duration before surgery was 45.8 months. The mean length of hospitalization was shorter in the MFx cohort (*p* = 0.01). No other difference was found between the two cohorts in terms of length of prior surgery symptoms and follow-up, mean age and BMI, sex and side, and defect size (Table 1).

### 3.3. Outcomes of Interest

At a mean follow-up of 43.1 months, the AOFAS (*p* = 0.03), VAS (*p* = 0.003), and Tegner (*p* = 0.01) scores were greater in the AMIC group. No difference was found in the MOCART score (*p* = 0.08) (Table 2).

### 3.4. Complications

The AMIC group experienced lower rates of reoperation (*p* = 0.008) and failure (*p* = 0.003). No hypertrophy or delamination were observed during follow-up (Table 3). Five patients (10%) underwent revision surgery for persistent pain within four years postoperatively in the AMIC group, seven (39%) in the MFx group. No complications related to the bone harvest site were experienced by any patient.

### 3.5. Subgroup Analysis

Within AMIC, no difference was evidenced by the subgroup analysis medial vs. lateral lesions (*p* = 0.08), bone grafting vs. no-bone grafting (*p* = 0.09), and between joint distractors vs. malleolar osteotomy (*p* = 0.07).

## 4. Discussion

According to the main findings of the present study, AMIC performed better than MFx at midterm follow-up. AOFAS and VAS both scored better in the AMIC group, along with a greater sporting activity, according to the Tegner score. The rate of reoperation and failure was also lower in the AMIC group at midterm follow-up. No complications related to the harvest site were reported. Within the AMIC group, no difference was evidenced by the subgroup analyses medial vs. lateral lesions, bone grafting vs. no-bone grafting, and joint distractors vs. malleolar osteotomy.

Symptomatic chondral defects negatively affect the activities of daily life and sporting activity level [25,31]. The low metabolic activity of hyaline cartilage, along with its alymphatic and hypocellular structure, are some of the reasons behind its poor regenerative capabilities [3,4,5]. Cartilage healing typically does not restore the original tissue, and fibrosis and residual chondral defects are common [32,33]. This impairs the cartilage biomechanical proprieties and predisposes to chronic pain [34,35]. AMIC has been successfully used to address chondral defects of the talus, with a growing trend of clinical studies in the current literature [36,37,38].

Only the study by Becher et al. [39] compared primary AMIC (N = 16) versus MFx (N = 16) for talar defects in a retrospective fashion; both techniques produced similar outcomes at five years follow-up. However, their patients had a mean defect smaller than 2 cm^2^ [39]. Chung et al. [40] compared AMIC versus MFx in 64 patients for knee chondral defects. They included patients with a mean defect size of 1.3 cm^2^ in the AMIC group and 1.5 cm^2^ in the MFx group, evidencing similarity between the two groups at two years follow-up. Both these studies were performed in a cohort of patients with small defects. Indeed, for small defects, MFx is still the most appropriate procedure [3,4,5,9]. Furthermore, the relatively short length of the follow-up may also affect the results. In the present study, we included only patients with borderline-sized defect (mean 2.7 cm^2^). We were unable to identify further studies that compare AMIC vs. MFx for talus chondral defects of this size. A similar study on chondral defects of the knee was performed by Volz et al. [41]. They compared AMIC versus MFx at five years in 47 patients with a mean defect size of 3.6 cm^2^. Similarly, they found a significant greater value of the Cincinnati score and lower pain level in the AMIC cohort.

We used plantar flexion and Hintermann spreader to distract the tibiotalar joint as standard, whereas, for lesions placed dorsally, a malleolar osteotomy was performed. Malleolar osteotomy leads to possible bony complications, intraoperative cartilage damage, loss of osteotomy reduction, delayed union or nonunion, persistent pain and/or swelling at the osteotomy site, painful hardware requiring surgical revision [42,43]. The osteotomy might damage the articular facet, and, given the poor regenerative capabilities of hyaline cartilage, this may lead to pain and early osteoarthrosis of the tibiotalar joint. Plantar flexion and Hintermann spreader to distract the tibiotalar allows patients to faster full bearing and recovery, but it may predispose to soft tissue damage, especially the neurovascular structures [44]. Indeed, in ankle arthroscopy series with mechanical distraction, the average complication rate ranges between 8 and 17% [45,46,47,48,49,50], while in a series of 1305 consecutive procedures with only plantar flexion the rate of complication was 3.4% [51]. In a cadaveric study, de Leeuw et al. [52] demonstrated greater distance between the anterior distal tibia and the overlying anterior neurovascular bundle with the ankle in a dorsiflexed position compared to the distracted ankle position. Whether plantar flexion and Hintermann spreader perform better than malleolar osteotomy is still controversial, and future studies are required.

The relatively small number of patients included in the present investigation represents the most important limitation of the present study, and may affect the ability to identify uncommon complications. The unblinded design, along with the lack of randomization, are two important limitations of the study. Further larger randomized controlled trials are required. We emphasize, however, that randomization and blinding in surgery may not be easily acceptable to patients and surgeons alike. The MOCART score was used to assess the degree of cartilage regeneration [53], but MRI is not reliable in predicting clinical outcome after cartilage repair is uncertain [54,55,56]. During the time elapsed between the onset of symptoms and the index surgery, all the patients underwent conservative management. However, given the heterogeneous nature and/or the lack of documentation of these treatments, it was not possible to analyze them separately. Similarly, most of the 15 patients who underwent revision surgeries were not aware of the treatment and/or data were lacking; thus, no further analyses were possible.

## 5. Conclusions

The present study confirmed our hypothesis that AMIC demonstrated superiority over MFx in focal osteochondral lesions of the talus between 2 and 3 cm^2^. At midterm follow-up, AOFAS and VAS scores were both better in the AMIC group, along with a greater sporting activity, according to the Tegner score. The rate of reoperation and failure was also lower in the AMIC group.

## Figures and Tables

**Table 1:** Demographic data of the patients (n.s.: not significant; AMIC: Autologous Matrix Induced Chondrogenesis; MFx: Microfracures; BMI: Body Mass Index).

Endpoint		AMIC (n = 52)	MFx (n = 18)	*p*
Follow-up (months)		44.2 ± 19.9	41.5 ± 18.1	n.s.
Mean age		31.5 ± 2.1	33.3 ± 6.2	n.s.
Sex (female)		44% (23 of 52)	44% (8 of 18)	n.s.
Right ankle		52% (27 of 52)	56% (10 of 18)	n.s.
Articular side (talus)				
medial		60% (31 of 52)	72% (13 of 18)	n.s.
lateral		40% (21 of 52)	18% (5 of 18)	n.s.
Cancellous bone grafting (n)		39% (20 of 52)		
from osteotomy site		14% (7 of 52)		
from iliac crest		25% (13 of 52)		
Approach				
Malleolar osteotomy (n)		44% (23 of 52)		
Distraction (n)		56% (29 of 52)		
Symptom duration (months)		48.1 ± 80.7	39.3 ± 50.41	n.s.
Length of stay (days)		3.5 ± 1.6	1.9 ± 2.0	0.01
Area of defect (cm^2^)		2.8 ± 1.5	2.4 ± 0.4	n.s.
BMI (kg/m^2^)		27.1 ± 6.4	26.9 ± 3.8	n.s.

**Table 2:** Results of scores (AMIC: Autologous Matrix Induced Chondrogenesis; MFx: Microfracures; VAS: Visual Analogue Scale; AOFAS: American Orthopedic Foot and Ankle Score; MOCART: Magnetic Resonance Observation of Cartilage Repair Tissue).

Endpoint	AMIC (n = 52)	MFx (n = 18)	MD	95% CI	*p*
MOCART	80.0 ± 25.4	66.8 ± 33.1	13.2	−1.822 to 28.222	0.08
AOFAS	83.8 ± 12.4	75.0 ± 19.3	8.8	0.921 to 16.679	0.03
VAS (0–10)	1.9 ± 0.8	3.3 ± 3.1	1.4	−2.326 to −0.474	0.003
Tegner	4.3 ± 1.5	3.1 ± 2.1	1.2	0.288 to 2.112	0.01

**Table 3:** Complications.

Endpoint	AMIC (n = 52)	MFx (n = 18)	OR	95% CI	*p*
Reoperation	10% (5 of 52)	39% (7 of 18)	0.17	0.0446 to 0.6271	0.008
Failures	13% (7 of 52)	50% (9 of 18)	0.16	0.0459 to 0.5268	0.003

## Data Availability

Data is contained within the article.
